# Inhibition of *Ref(2)P*, the Drosophila homologue of the *p62/SQSTM1* gene, increases lifespan and leads to a decline in motor function

**DOI:** 10.1186/s13104-021-05462-6

**Published:** 2021-02-08

**Authors:** Emily P. Hurley, Brian E. Staveley

**Affiliations:** Department of Biology, Memorial University of Newfoundland, St. Johns’, NL A1B 3X9 Canada

**Keywords:** *Drosophila melanogaster*, *p62*, *Sequestosome1*, *Ref(2)P*, Longevity, Locomotor decline, *Drosophila* models of neurodegenerative disease

## Abstract

**Objective:**

Sequestosome 1 (p62/SQSTM1) is a multifunctional scaffold/adaptor protein encoded by the *p62/SQSTM1* gene with function in cellular homeostasis. Mutations in the *p62/SQSTM1* gene have been known to be associated with patients with amyotrophic lateral sclerosis (ALS), frontotemporal dementia (FTD), and Parkinson disease (PD). The aim of the present study was to create a novel model of human neurogenerative disease in *Drosophila melanogaster* by altering the expression of *Ref(2)P*, the Drosophila orthologue of the human *p62/SQSTM1* gene. *Ref(2)P* expression was altered in all neurons, the dopaminergic neurons and in the motor neurons, with longevity and locomotor function assessed over time.

**Results:**

Inhibition of *Ref(2)P* resulted in a significantly increased median lifespan in the motor neurons, followed by a severe decline in motor skills. Inhibition of *Ref(2)P* in the dopaminergic neurons resulted in a significant, but minimal increase in median lifespan, accompanied by a drastic decline in locomotor function. Inhibition of *Ref(2)P* in the *ddc-Gal4*-expressing neurons resulted in a significant increase in median lifespan, while dramatically reducing motor function.

## Introduction

The elucidation of the cellular mechanisms that are altered during the progression of neurodegenerative diseases, such as amyotrophic lateral sclerosis (ALS) and Parkinson disease (PD), is an ongoing subject of current research. The protein, sequestosome1, which is also known as p62 (*p62/SQSTM1*), has been suggested to be a potential contributor to in the pathogenesis of a number of neurodegenerative diseases [1]. The p62/SQSTM1 protein is a multifunctional scaffold/adaptor protein encoded by the *p62/SQSTM1* gene [2]. Alternatively designated as Refractory to Sigma P” (*Ref(2)P*) in Drosophila, p62/SQSTM1 is involved in various aspects of selective autophagy—such as mitophagy, the ubiquitin–proteasome system (UPS) and in some signal transduction pathways [3]. The p62/SQSTM1 protein is localized throughout the cell in the cytoplasm, in the cytosol, and the endoplasmic reticulum, among other places such as autophagosomes, aggresomes, and autolysosomes, as this protein functions during the process of autophagy [4, 5]. The role which *p62/SQSTM1* has in autophagy is critical as it seems to regulates the removal of protein aggregates and damaged organelles through the activities of several of its many functional domains [3]. Structurally the p62/SQSTM1 protein has many functional domains, several of which are essential to autophagic activities. These domains include the Phox and Bem1 (PB1) domain, the LC3 interacting region (LIR) domain, and the UBA domain at the C-terminus [6]. During autophagy, the LIR domain and the UBA domains of *p62/SQSTM1* have vital roles in the pathway [1, 7, 8], as *p62/SQSTM1* promotes autophagic degradation by binding to LC3 via its LIR region [9]. The PB1 and the UBA domains function together to form protein aggregates [10], and are known to be critical for mitochondrial clustering [11]. Not only does *p62/SQSTM1* function in quality control through roles in autophagy, mitophagy and UPS, but it is known to have roles in age-related diseases. Mutations in *p62/SQSTM1* are known to be associated with ALS and frontotemporal dementia (FTD) [2], as well as with some forms of Parkinson disease through a role in mitophagy [12]. Where during PINK1-parkin-mediated mitophagy, p62/SQSTM1 among other adaptor proteins are recruited to damaged mitochondria [13]. Studies in Drosophila have demonstrated that loss-of-function of *Ref(2)P* results in poor locomotor function related to mitochondrial dysfunction and accumulation of mitochondrial DNA [11, 14]. Through the investigation of *p62/SQSTM1* as a candidate gene may further insight into our understanding of *p62/SQSTM1* function in both ageing and neurodegenerative disease. It was predicted that inhibiting the *Ref(2)P* gene in Drosophila would impair median organism lifespan and locomotor ability, as the loss-of-function mutations in the orthologous *p62/SQSTM1* gene have been observed in ALS, FTD and PD patients. The aim of the present study was to create a *p62/SQSTM1*-based model of human neurodegenerative disease in *D. melanogaster* by altering the expression of the orthologous *Ref(2)P* gene.

## Materials and methods

### Drosophila stocks and culture

The *P{y[*+ *t7.7] v[*+ *t1.8]* = *TRiP.HMS00551}attP2* (*UAS-Ref(2)P-RNAi *^*HMS00551*^), *P{y[*+ *t7.7] v[*+ *t1.8]* = *TRiP.HMS00938}attP2* (*UAS-Ref(2)P-RNAi*^*HMS00938*^), and *P{UAS-lacZ.B}meltBg4-1–2* (*UAS-lacZ*) responder lines as well as the *Gal4* lines *D42-Gal4*, *TH-Gal4*, *ddc-Gal4*^*HL4.3D*^, were all obtained from the Bloomington Drosophila Stock Center at Indiana University (IN, USA). *UAS-LacZ* has been used as a benign control in a number of experiments produced in our lab as other controls investigated to date do not have a benign response and may reduce median lifespan. *Drosophila melanogaster* was maintained on a standard media comprised of 65 g/L cornmeal, 50 ml/L fancy grade molasses, 10 g/L yeast and 5.5 g/L agar which was then treated with 2.5 ml propionic acid and 5 ml of 0.1 g/ml methylparaben. This mixture was then allowed to solidify at the bottom of vials and stored in 4 to 6 °C until use. Stocks were stored at room temperature (~ 21 °C), while crosses and experiments were performed at 25 °C. Quantification of inhibition efficiency was not conducted in this study, however, the *UAS-Ref(2)P-RNAi *^*HMS00551*^ has been quantified by Liu et al. (2018).

### Drosophila crosses

Virgin female flies from the *D42-Gal4*, *TH-Gal4*, *ddc-Gal4*^*HL4.3D*^ lines were bred with male flies which expressed *Ref(2)P* inhibition. The consistent material and paternal sources allow for minimal genetic differences. Two *Ref(2)P* inhibition lines *UAS-Ref(2)P-RNAi *^*HMS00551*^, *UAS-Ref(2)P-RNAi*^*HMS00938*^ and the control line *UAS-lacZ* were used. Critical class male progeny from these crosses were assessed for longevity and locomotor ability through ageing and climbing assays. While UAS-LacZ does not produce a non-silencing RNAi control it is still a valid control for the GAL4 and responding transgenes. Future experiments may include the addition of a RNAi-silencing control if shown to be reasonable.

### Longevity assays

The survival of Drosophila was analyzed to examine the lifespan of experimental flies in comparison to control flies. Critical class male progeny were collected daily and placed in vials with fresh medium. A sample size of approximately three hundred males was collected in total and stored at 25 °C for the duration of the experiment. The flies were scored every two days to examine if any death had occurred. A fly was considered dead when no movement was observed. Males were transferred onto fresh media every 4 days to obtain a healthy environment. Graphpad Prism 8 (Graphpad Software Inc.) was used to analyze longevity data, and survival curves were analyzed and compared using the Log-rank (Mantel-Cox) test, with a *P*-value less than or equal to 0.05 with Bonferroni correction being considered statistically significant. Longevity data is a representation of the summation of independent replicates.

### Locomotor assays

Approximately 70 critical class male progeny to be collected from crosses between female *D42-Gal4*, *TH-Gal4*, and *ddc-Gal4*^*HL4.3D*^ flies and male *UAS-Ref(2)P-RNAi *^*HMS00551*^, *UAS-Ref(2)P-RNAi*^*HMS00938*^ and *UAS-lacZ* flies. This assay was used to measure the ability of flies to climb up a narrow glass tube over the course of their lifespan, with 50 male flies from each genotype being evaluated once every 7 days, beginning at the 7th day post-eclosion. Climbing analysis was conducted until flies had a minimum climbing score for 2 consecutive weeks, or less than 10 flies remained alive for that genotype. Class males were maintained in vials with ten flies per vial, stored at 25 °C, and placed on new medium once per week throughout the experiment. Climbing analysis followed the standard protocol outlined by our laboratory. Graphpad Prism 8 (Graphpad Software Inc.) was used to analyze the data, and to generate climbing curves fitted using non-linear regression. 95% confidence intervals were used to test for significance with the curves considered to be significantly different if *P* < 0.05. Locomotive data is a representation of the summation of independent replicates.

## Results

### Inhibition of *Ref(2)P* in the motor neurons increases longevity and reduces locomotor ability

**Fig. 1:**
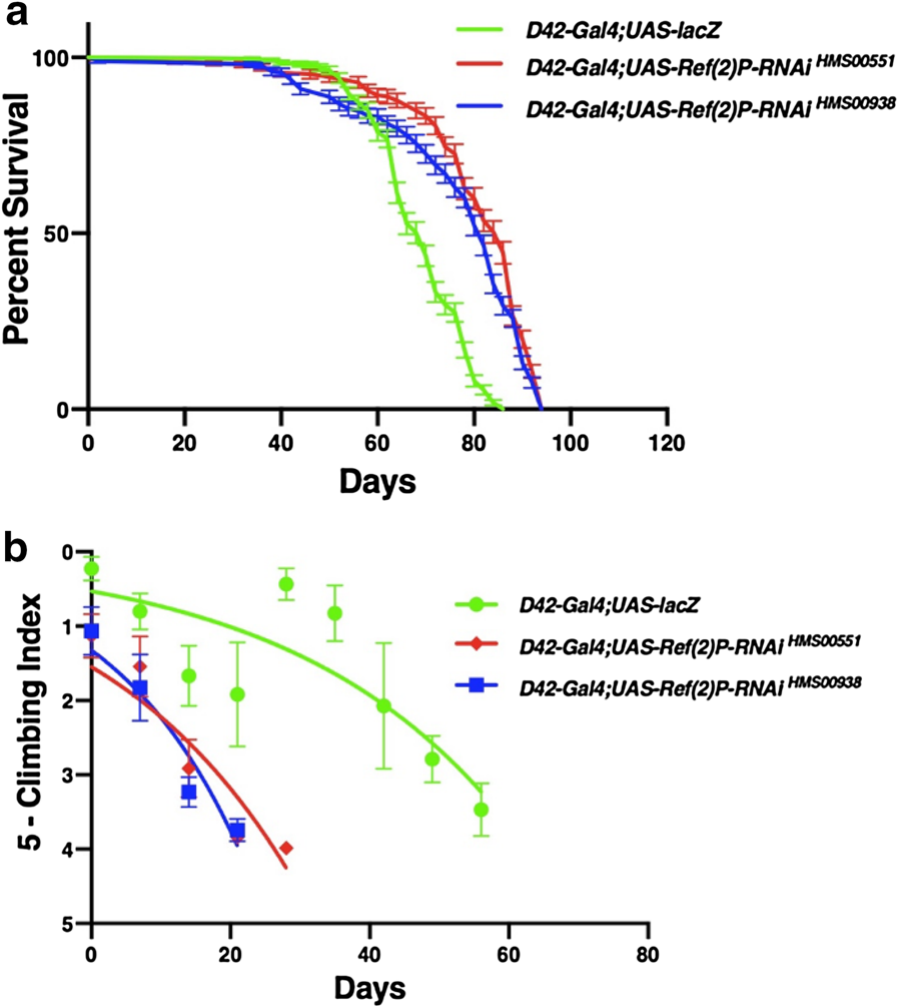
Altered expression of *Ref(2)P* directed through the *D42-Gal4* transgene affects longevity and climbing ability. **a** Longevity assay of *Drosophila melanogaster* males displaying altered *Ref(2)P* expression in the motor neurons. Longevity is depicted by percent survival. Significance is *P* < 0.05 using the log-rank test with Bonferroni correction. Error bars represent standard error of the mean. **b** Locomotor assay of *D. melanogaster* males displaying altered *Ref(2)P* expression in the motor neurons. Climbing ability was determined by a nonlinear curve fit (CI = 95%). Error bars indicate standard error of the mean

The inhibition of *Ref(2)P* via the *UAS-Ref(2)P-RNAi *^*HMS00551*^ transgene through *D42-Gal4* resulted in a significant increase in median lifespan by approximately 22%. Compared to the control *UAS-lacZ* median lifespan of 70 days, inhibition of *Ref(2)P* via *Ref(2)P-RNAi *^*HMS00551*^ lead to a median lifespan of 86 days (Fig. 1a). Locomotor ability was reduced over time compared to the control *UAS-lacZ* which maintained strong climbing ability well into the 8 weeks the *Ref(2)P-RNAi*^*HMS00551*^ flies lost their ability to climb at week 4 (Fig. 1b). Similarly, the inhibition of *Ref(2)P* via the *UAS-Ref(2)P-RNAi*^*HMS00938*^ transgene through *D42-Gal4* resulted in a significant increase in median lifespan by approximately 17%. Compared to the control *UAS-lacZ* median lifespan of 70 days, inhibition of *Ref(2)P* via *Ref(2)P-RNAi*^*HMS00938*^ lead to a median lifespan of 82 days (Fig. 1a). Locomotor ability was reduced over time compared to the control *UAS-lacZ* which maintained strong climbing ability well into the 8 weeks the *Ref(2)P-RNAi*^*HMS00938*^ flies lost their ability to climb at week 3 (Fig. 1b).

### Inhibition of *Ref(2)P* in the dopaminergic neurons increases longevity and reduces locomotor ability

**Fig. 2:**
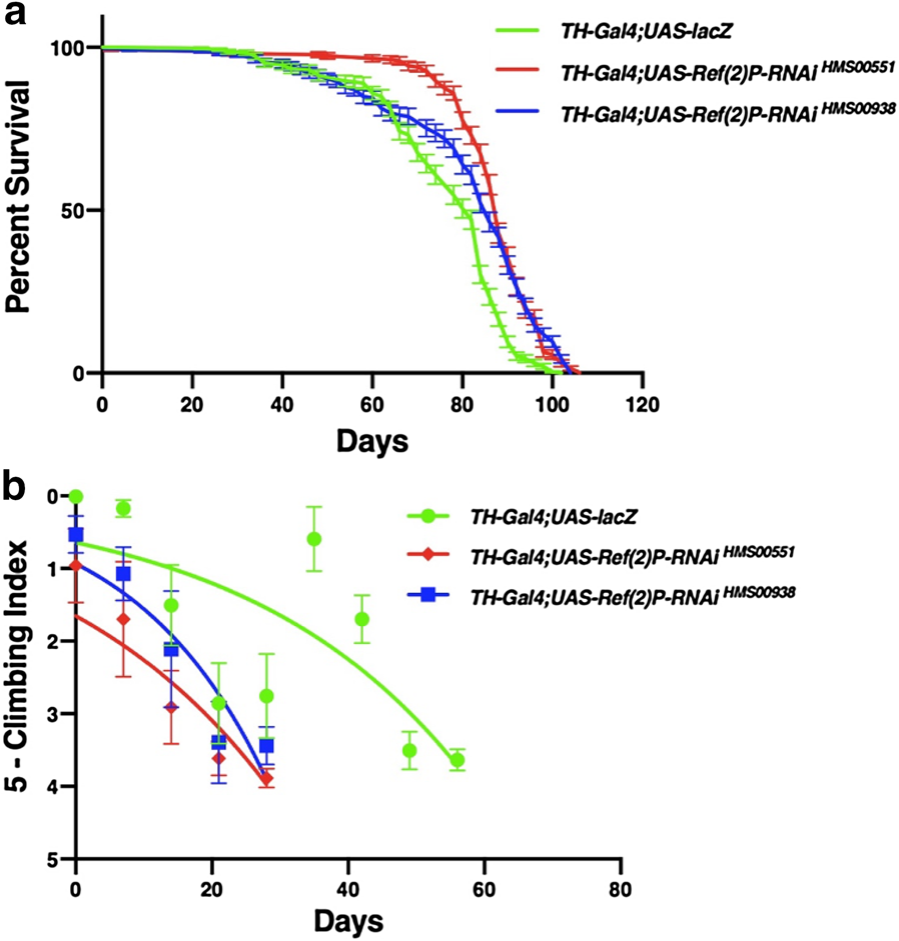
Altered expression of *Ref(2)P* through the *TH-Gal4* transgene affects longevity and climbing ability. **a** Longevity assay of *Drosophila melanogaster* males displaying altered *Ref(2)P* expression in the dopaminergic neurons. Longevity is depicted by percent survival. Significance is *P* < 0.05 using the log-rank test with Bonferroni correction. Error bars represent standard error of the mean. **b** Locomotor assay of *D. melanogaster* males displaying altered *Ref(2)P* expression in the dopaminergic neurons. Climbing ability was determined by a nonlinear curve fit (CI = 95%). Error bars indicate standard error of the mean

The inhibition of *Ref(2)P* via the *UAS-Ref(2)P-RNAi *^*HMS00551*^ transgene through *TH-Gal4* resulted in a significant increase in median lifespan compared to the control *UAS-lacZ* median lifespan of 82 days, inhibition of *Ref(2)P* via *Ref(2)P-RNAi *^*HMS00551*^ lead to a median lifespan of 88 days (Fig. 2a). Locomotor ability was reduced over time compared to the control *UAS-lacZ* which maintained strong climbing ability well into the 8 weeks the *Ref(2)P-RNAi *^*HMS00551*^ flies lost their ability to climb at week 4 (Fig. 2b). Similarly, the inhibition of *Ref(2)P* via the *UAS-Ref(2)P-RNAi*^*HMS00938*^ transgene through *TH-Gal4* resulted in a significant increase in median lifespan compared to the control *UAS-lacZ* median lifespan of 82 days, inhibition of *Ref(2)P* via *Ref(2)P-RNAi*^*HMS00938*^ lead to a median lifespan of 86 days (Fig. 2a). Locomotor ability was reduced over time compared to the control *UAS-lacZ* which maintained strong climbing ability well into the 8 weeks the *Ref(2)P-RNAi*^*HMS00938*^ flies lost their ability to climb at week 4 (Fig. 2b).

### Inhibition of *Ref(2)P* in the *ddc-Gal4*^*HL4.3D*^-expressing neurons increases longevity and reduces locomotor ability

**Fig. 3:**
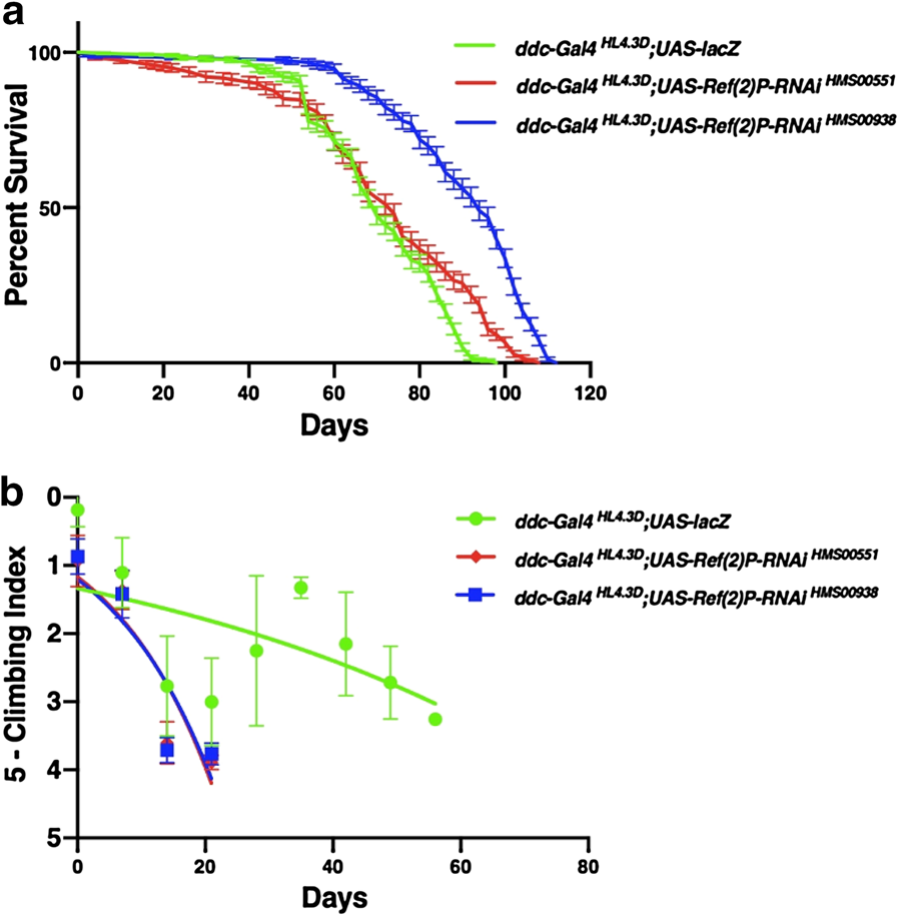
Altered expression of *Ref(2)P* directed through the *ddc-Gal4*^*HL4.3D*^ transgene affects longevity and climbing ability. **a** Longevity assay of *Drosophila melanogaster* males displaying altered *Ref(2)P* expression in the motor neurons. Longevity is depicted by percent survival. Significance is *P* < 0.05 using the log-rank test with Bonferroni correction. Error bars represent standard error of the mean. **b** Locomotor assay of *D. melanogaster* males displaying altered *Ref(2)P* expression in the motor neurons. Climbing ability was determined by a nonlinear curve fit (CI = 95%). Error bars indicate standard error of the mean

The inhibition of *Ref(2)P* via the *UAS-Ref(2)P-RNAi *^*HMS00551*^ transgene through *ddc-Gal4*^*HL4.3D*^ resulted in a significant increase in median lifespan compared to the control *UAS-lacZ* median lifespan of 70 days, inhibition of *Ref(2)P* via *Ref(2)P-RNAi *^*HMS00551*^ lead to a median lifespan of 74 days (Fig. 3a). Locomotor ability was reduced over time compared to the control *UAS-lacZ* which maintained strong climbing ability well into the 8 weeks the *Ref(2)P-RNAi *^*HMS00551*^ flies lost their ability to climb at week 4 (Fig. 3b). The inhibition of *Ref(2)P* via the *UAS-Ref(2)P-RNAi*^*HMS00938*^ transgene through *ddc-Gal4*^*HL4.3D*^ resulted in a significant increase in median lifespan by approximately 34%. Compared to the control *UAS-lacZ* median lifespan of 70 days, inhibition of *Ref(2)P* via *Ref(2)P-RNAi*^*HMS00938*^ lead to a median lifespan of 94 days (Fig. 3a). Locomotor ability was reduced over time compared to the control *UAS-lacZ* which maintained strong climbing ability well into the 8 weeks the *Ref(2)P-RNAi*^*HMS00938*^ flies lost their ability to climb at week 4 (Fig. 3b).

## Discussion

The *p62/SQSM1* gene is known to be associated with the human neurodegenerative disease ALS, FTD, and more recently in Parkinson Disease. We found that the inhibition of the *Ref(2)P* gene results in a significant increase in median lifespan, accompanied by a severe reduction in locomotor function over time. The inhibition of *Ref(2)P* through the expression of *UAS-Ref(2)P-RNAi *^*HMS00551*^ in the motor neurons gave a significant increase in median lifespan by approximately 22%, while resulting in a drastic decrease in locomotor function over time. Similarly, the inhibition of *Ref(2)P* through the expression of *UAS-Ref(2)P-RNAi*^*HMS00938*^ in the motor neurons gave a significant increase in median lifespan by approximately 17%, while severely reducing locomotor ability over time. The increase in lifespan accompanied by a sharp decrease in motor function over time may be interpreted as trade-off, where the slight increase in longevity is a type of compensation for a severe decline in motor skills. Inhibiting *Ref(2)P* in the motor neuron was conducted as both ALS and Parkinson Disease are known to result in a loss of motor function, with ALS being a motor-neuron disease. This significant reduction in motor ability and increase in lifespan when *Ref(2)P* expression is altered corresponds with the characteristic loss of motor neurons associated with ALS, making the inhibition of *Ref(2)P* in the motor neuron an imperfect model of neurodegenerative disease.

The inhibition of *Ref(2)P* through the expression of *UAS-Ref(2)P-RNAi *^*HMS00551*^ in the dopaminergic neurons gave a significant increase in median lifespan, while resulting in a drastic decrease in locomotor function over time. Similarly, the inhibition of *Ref(2)P* through the expression of *UAS-Ref(2)P-RNAi*^*HMS00938*^ in the dopaminergic neurons gave a significant increase in median lifespan, while severely reducing locomotor ability over time. Similar to the inhibition of *Ref(2)P* in the motor neurons, the minimal increase in lifespan and drastic reduction in motor skills seen under the inhibition of *Ref(2)P* in the dopaminergic neurons can be interpreted as a type of compensation. As dopaminergic transmission is known to be altered during Parkinson Disease pathology, investigating the consequences of *Ref(2)P* in the dopaminergic neurons would be of great interest. This significant reduction in motor ability and minimal increase in lifespan seen when *Ref(2)P* expression is altered, corresponds with the characteristic loss of dopaminergic neurons associated with Parkinson Disease, making the inhibition of *Ref(2)P* in the motor neuron an promising model of neurodegenerative disease.

The inhibition of *Ref(2)P* through the expression of *UAS-Ref(2)P-RNAi *^*HMS00551*^ in the *ddc-Gal4*^*HL4.3D*^-expressing neurons gave a significant increase in median lifespan, while dramatically reducing locomotor ability over time. The *ddc-GAL4-*expressing neurons include both dopaminergic and serotonergic neurons, as dopaminergic transmission is known to play a role in Parkinson Disease investigating the consequences of Ref(2)P inhibition in these neurons was conducted. This significant reduction in motor ability and minimal increase in lifespan seen when *Ref(2)P* expression is altered suggest that the inhibition of *Ref(2)P* in the *ddc-Gal4*^*HL4.3D*^-expressing neurons to be a promising model of neurodegenerative disease. On the other hand, the inhibition of *Ref(2)P* through the expression of *UAS-Ref(2)P-RNAi*^*HMS00938*^ in the *ddc-Gal4*^*HL4.3D*^-expressing neurons gave a significant increase in median lifespan by approximately 34%, accompanied by a severe reduction locomotor ability over time. This significant reduction in motor ability and increase in lifespan seen when *Ref(2)P* expression is altered may also be interpreted as a type of compensation, and seen as an imperfect model of neurodegenerative disease.

Investigating the inhibition of *Ref(2)P* expression in a tissue-specific manner demonstrates the phenotypic consequences to the organism in a more direct manner. By inhibiting expression in the motor neuron, dopaminergic neuron and *ddc-GAL4*-expressing neurons, rather than the generating mutant *Ref(2)P* critical class which express *Ref(2)P* inhibition throughout the whole body, less restrictive results are produced that show the consequences of tissue-specific loss-of-function. While the loss-of-function of *Ref(2)P* has shown a reduction in climbing ability, which can be a promising model of neurodegenerative disease, but yet increase in median lifespan, this cannot be explained through this study, and instead can be seen as a compensational trade-off.

### Limitations

To significantly add to our experimental design, the directed overexpression of *Ref(2)P* would be highly desirable and essential for a “gene replacement” study of *Ref(2)P*. Although several *Gal-4* transgenes were employed in this set of experiments, a number of others could prove to informative. Our future plans include combining the inhibition of *Ref(2)P* with other gene manipulations to identify interactions including synergies that further increase lifespan and improve locomotor activity over time. Although UAS-LacZ is not the best control it has been used as benign control in our lab, we aim in the future to explore other controls that may be better. Finally, as critical class males were analysed, broadening the study to critical females might be very provide useful data.

## Data Availability

Data analyzed during this study are included in the article figures.

## Abbreviations

ALS: Amyotrophic lateral sclerosis
Ddc: Dopa decarboxylase
PD: Parkinson disease
RNAi: Ribonucleic acid interference
*Ref(2)P*: Refractory to sigma P
TH: Tyrosine hydroxylase

## References

[1] Ma S, Attarwala IY, Xie X (2019). SQSTM1 / p62: a potential target for neurodegenerative disease [review-article]. ACS Chem Neurosci.

[2] Bartolome F, Esteras N, Martin-requero A, Boutoleau C (2017). Impair energy metabolism through limitation of mitochondrial substrates. Sci Rep.

[3] Bitto A, Lerner CA, Nacarelli T, Crowe E, Torres C, Sell C (2014). p62/SQSTM1 at the interface of aging, autophagy, and disease. Age.

[4] Liu WJ, Ye L, Huang WF, Guo LJ, Xu ZG, Wu HL, Liu HF (2016). P62 links the autophagy pathway and the ubiqutin-proteasome system upon ubiquitinated protein degradation. Cell Mol Biol Lett.

[5] Matsumoto G, Shimogori T, Hattori N, Nukina N (2015). TBK1 controls autophagosomal engulfment of polyubiquitinated mitochondria through p62/SQSTM1 phosphorylation. Hum Mol Genet.

[6] Liang X, Guan X (2017). Frontiers in Laboratory Medicine p62 / SQSTM1: a potential molecular target for treatment of atherosclerosis. Front Lab Med.

[7] Hou B, Wang G, Gao Q, Wei Y, Zhang C, Wang Y (2019). SQSTM1 / p62losreverses the inhibitory effect of sunitinib on autophagy independent of AMPK signaling. Sci Rep.

[8] Johansen T, Lamark T (2011). Selective autophagy mediated by autophagic adapter proteins. Autophagy.

[9] Pankiv S, Hoyvarde Clausen T, Lamark T, Brech A, Brunn J-A, Outzen H, Johansen T (2007). p62 / SQSTM1 binds directly to Atg8 / LC3 to facilitate degradation of ubiquitinated protein aggregates. J Biol Chem.

[10] Bartlett BJ, Isakson P, Lewerenz J, Sanchez H, Kotzebue RW, Cumming R, Finley KD (2011). p62, Ref(2)P and ubiquitinated proteins are conserved markers of neuronal aging, aggregate formation and progressive autophagic defects. Autophagy.

[11] De Castro IP, Costa AC, Celardo I, Tufi R, Dinsdale D, Loh SHY, Martins LM (2013). Drosophila ref (2)P is required for the parkin -mediated suppression of mitochondrial dysfunction in pink1 mutants. Cell Death Dis.

[12] Narendra DP, Kane LA, Hauser DN, Fearnley IM, Youle RJ (2010). p62/SQSTM1 is required for Parkin-induced mitochondrial clustering but not mitophagy; VDAC1 is dispensable for both. Autophagy.

[13] Xiao B, Deng X, Lim GGY, Zhou W, Saw W, Dong Z, Tan E (2017). BBA molecular cell research p62-mediated mitochondrial clustering attenuates apoptosis induced by mitochondrial depolarization. BBA Mol Cell Res.

[14] de Castro IP, Costa AC, Lam D, Tufi R, Fedele V, Moisoi N, Martins LM (2012). Genetic analysis of mitochondrial protein misfolding in *Drosophila melanogaster*. Cell Death Differ.

